# Plants, Lichens, Fungi, and Algae Extracts and Derivatives with Antimicrobial Properties for Nutrition and Health

**DOI:** 10.3390/antibiotics15010111

**Published:** 2026-01-22

**Authors:** Violeta Popovici, Emma Adriana Ozon, Andreea Letiția Arsene, Verginica Schröder

**Affiliations:** 1 Center for Mountain Economics, “Costin C. Kiriţescu” National Institute of Economic Research (INCE-CEMONT), Romanian Academy, 725700 Vatra-Dornei, Romania; 2 Department of Pharmaceutical Technology and Biopharmacy, Faculty of Pharmacy, “Carol Davila” University of Medicine and Pharmacy, 020956 Bucharest, Romania; emma.budura@umfcd.ro; 3 Department of Pharmaceutical Microbiology, Faculty of Pharmacy, “Carol Davila” University of Medicine and Pharmacy, 020956 Bucharest, Romania; andreea.arsene@umfcd.ro; 4 Department of Cellular and Molecular Biology, Faculty of Pharmacy, Ovidius University of Constanta, 6 Capitan Al. Serbanescu Street, 900001 Constanta, Romania; verginica.schroder@univ-ovidius.ro

Plants are a rich source of bioactive compounds with a wide range of nutritional and therapeutic properties [1]. Studies have demonstrated their potential to prevent and treat various infectious diseases, highlighting the importance of their phytochemicals as antioxidants and antimicrobial agents. Plant-derived compounds present a promising opportunity to combat a wide range of bacterial pathogens, including those prioritized by the World Health Organization (WHO) [2]. Their unique chemical structures, often difficult or impossible to replicate with synthetic methods, highlight the potential of natural compounds to address bacterial resistance [3]. By leveraging various mechanisms to target microbial pathogens, these compounds showcase a multifaceted approach to anti-infectious therapy [4]. Furthermore, their structural diversity, combined with their safety, underscores the importance of further exploring natural substances in the development of effective antimicrobial therapies [5].

This Special Issue focuses on the above-mentioned topics by presenting the most recent research on the antimicrobial activity of various plant species and plant-derived products. New and valuable data have been added to this field by the authors of the nine original studies in this Special Issue.

Plant extracts are widely studied for their inhibitory activity against various pathogens. Unlike conventional antibiotics, which often target a single site (leading to resistance), plant extracts exhibit multiple mechanisms, depending on the phytochemicals extracted and the solvents used (mainly water or ethanol) [6]. Lipophilic compounds disrupt cell membrane integrity, leading to the leakage of cellular contents [7]. Phenolic compounds bind to bacterial enzymes and cell wall proteins, thereby neutralizing microbial function and replication [8]. Lectins are valuable agents in preventing the attachment of viruses and bacteria to host cells [9,10,11]. Other plant extracts can alter the cytoplasmic pH of microorganisms, thereby disrupting their metabolic processes [12]. These mechanisms offer a promising avenue for further exploration in microbial management and the development of treatment protocols.

Three original studies examined the phytochemical composition and pharmacological potential of different plant extracts in ethanol.

First, an interdisciplinary Romanian research team conducted a complex analysis of the phenolic profile and selected biological properties of ethanolic extracts of *Agave amica* (Medik.) Thiede & Govaerts cultivated in Romania [13]. This research is a comparative study of the polyphenolic profile and the antioxidant, antimicrobial, antibiofilm, and cytotoxic/antiproliferative properties of 70% ethanolic extracts from the aerial parts and bulbs of *A. amica.* Both ethanolic extracts had high polyphenol content, with caffeic acid identified as a key compound. The aerial part extract shows promising results, with significantly higher concentrations of total polyphenols, tannins, flavonoids, and caffeic acid derivatives, as well as greater antioxidant activity than the bulb extract (*p* < 0.001). Both extracts exhibited significant inhibition of *Candida albicans* growth and moderate efficacy against *Listeria monocytogenes* and *Staphylococcus aureus*, affecting both planktonic cells and biofilms. Furthermore, promising evidence of dose-dependent cytotoxicity against the colorectal adenocarcinoma cell line suggests pathways for further research on potential therapeutic applications [13].

In the second paper, researchers from Thailand investigated the ethanolic extracts of Thai medicinal flowers—*Mesua ferrea* L. (Bunnak), *Mammea siamensis* T. Anderson (Saraphi), and *Clitoria ternatea* (Anchan)—known for their use in traditional medicine. The phytochemical profile determined by HPLC indicated that the *M. ferrea* extract contained the highest level of gallic acid. In contrast, the *M. siamensis* extract reported the highest quercetin concentration. LC-MS analysis identified fifteen key phenolic metabolites in all Thai flower extracts. The extract from *C. ternatea* had the highest total flavonoid content and showed substantial antioxidant activity. Antibacterial activity against enteric pathogens (*E. coli*, *E. coli* O157:H7, *Salmonella typhi*, *Shigella dysenteriae,* and *Vibrio cholerae*) revealed that M. ferrea exhibited the most significant inhibitory effects. All extracts exhibited time-dependent antibacterial activity, significantly prevented biofilm formation, disrupted established biofilms, reduced bacterial adhesion to intestinal cells, and induced membrane damage in *E. coli* O157:H7, leading to leakage of cellular components (DNA, RNA, and proteins). The second study results recommend Thai flower extracts as plant-based alternatives for preventing or controlling enteric bacterial infections [14].

The third paper reported the antiviral (anti-HIV), antifungal (anti-*C. albicans*), and antibacterial (anti-*E. coli*) activities of a green ultrasound-assisted extract from different parts of *Momordica charantia* fruits cultivated in India and Saudi Arabia. The authors identified and quantified, using LC-MS/MS analysis, three bioactive cucurbitane triterpenoid glycosides (karavilosides) in the *M. charantia* extracts tested, which are responsible for their pharmaceutical potential, and supported their results with molecular docking [15].

Incorporating plant extracts into nanoparticles (NPs) is an innovative approach to enhancing antibacterial activity. By combining bioactive plant phenolic metabolites with the advantageous properties of nanoparticles—such as their large surface area and targeted delivery capabilities—potent antimicrobial agents can be developed. These nanoparticles, which often include silver, zinc oxide, or copper oxide, show great promise for combating drug-resistant bacteria such as *E. coli* and *S. aureus.* They have effective antibacterial mechanisms (disrupting bacterial cells and generating reactive oxygen species (ROS)) and could be a cost-effective alternative to traditional antibiotics [16]. Thus, Jeon et al. synthesized silver nanoparticles (AgNPs) using an extract of *Morus nigra* L. [17]. The green-synthesized nanoparticles exhibited high antioxidant, cytotoxic, and anti-inflammatory activities, and significantly increased inhibitory effects against *E. coli* and *S. aureus* [17].

Propolis is a natural product derived from three primary sources: plant resins collected by bees, substances produced through their metabolic processes (including wax), and additional materials incorporated during propolis processing. The chemical composition of propolis is strongly determined by the specific plant sources of the resins, making it a unique and valuable plant-derived product with antimicrobial properties [18]. Thus, Oliveira et al. evaluated the inhibitory effects of hydroethanolic extracts of Portuguese propolis harvested from the Gerês apiary against a panel of three Gram-positive (*Bacillus subtilis*, methicillin-sensitive *S. aureus* (MSSA), and methicillin-resistant *S. aureus* (MRSA)) and one Gram-negative bacterium (*E. coli*), as well as two yeasts (*C. albicans* and *Saccharomyces cerevisiae*). The authors compared the antibacterial activity of propolis extracts with that of three conventional antibiotics (erythromycin, vancomycin, and amoxicillin/clavulanate). They found that propolis exhibited greater antimicrobial activity against *B. subtilis* than vancomycin. Moreover, MRSA strains were resistant to all three broad-spectrum antibiotics and susceptible to propolis extracts [19].

Essential oils exhibit antimicrobial activity through various mechanisms that contribute to their effectiveness. These include disrupting the structure of cell walls, which enhances permeability, and altering the flow of electrons and protons. Additionally, they promote active transport, facilitate the coagulation of cell contents, damage cytoplasmic membranes, and hydrolyze ATP. By understanding these mechanisms, we can better harness the antimicrobial potential of essential oils, which can synergize with conventional antibiotics [20,21]. Four original studies that analyzed the inhibitory activity of various essential oils against food-borne and other clinically significant pathogens were published in our Special Issue. The results obtained by research teams from Italy, Romania, Poland, Turkey, and Portugal could enrich the scientific database with valuable information regarding the antimicrobial activity and potential synergism of the essential oils investigated in the contributions listed below.

1. Iseppi, R.; Truzzi, E.; Sabia, C.; Messi, P. Efficacy and Synergistic Potential of Cinnamon (*Cinnamomum zeylanicum*) and Clove (*Syzygium aromaticum* L. Merr. & Perry) Essential Oils to Control Food-Borne Pathogens in Fresh-Cut Fruits. *Antibiotics* **2024**, *13*, 319. https://doi.org/10.3390/antibiotics13040319
2. Neagu, R.; Popovici, V.; Ionescu, L.-E.; Ordeanu, V.; Biță, A.; Popescu, D.M.; Ozon, E.A.; Gîrd, C.E. Phytochemical Screening and Antibacterial Activity of Commercially Available Essential Oils Combinations with Conventional Antibiotics against Gram-Positive and Gram-Negative Bacteria. *Antibiotics* **2024**, *13*, 478. https://doi.org/10.3390/antibiotics13060478
3. Luca, S.V.; Wojtanowski, K.; Korona-Głowniak, I.; Skalicka-Woźniak, K.; Minceva, M.; Trifan, A. Spent Material Extractives from Hemp Hydrodistillation as an Underexplored Source of Antimicrobial Cannabinoids. *Antibiotics* **2024**, *13*, 485. https://doi.org/10.3390/antibiotics13060485
4. Kılıç, C.S.; Catarino, I.; Alves-Silva, J.; Demirci, B.; Kırcı, D.; Salgueiro, L.; Zuzarte, M. Unlocking the Skin-Protective Effects of *Ferulago* spp. Essential Oils: A Focus on Antidermatophytic and Wound-Healing Potential. *Antibiotics* **2025**, *14*, 343. https://doi.org/10.3390/antibiotics14040343
